# Late Postoperative Capsular Bag Distension Syndrome Presenting 15 Years After Surgery Successfully Treated With Neodymium-Doped Yttrium Aluminum Garnet Posterior Capsulotomy

**DOI:** 10.7759/cureus.111770

**Published:** 2026-06-29

**Authors:** Jonathan D Shirian, Nadim S Azar, Meghan M Brown, Amy Lin

**Affiliations:** 1 School of Medicine, Case Western Reserve University, Cleveland, USA; 2 John A. Moran Eye Center, University of Utah, Salt Lake City, USA

**Keywords:** capsular bag distension syndrome, capsular block syndrome, cataract surgery complications, nd:yag capsulotomy, posterior capsule opacification (pco)

## Abstract

A 74-year-old man presented with progressively worsening hazy vision in his right eye 15 years after cataract surgery. He had previously undergone an unsuccessful neodymium-doped yttrium aluminum garnet (Nd:YAG) posterior capsulotomy elsewhere for presumed posterior capsule opacification. Slit lamp examination revealed yellow turbid fluid distending the capsular bag behind the intraocular lens with posterior bowing of the posterior capsule, consistent with late postoperative capsular bag distension syndrome. No significant anterior chamber inflammation was observed. A repeat Nd:YAG posterior capsulotomy was performed using an Abraham lens, resulting in immediate decompression of the capsular bag. The intraocular lens remained well centered, and the patient reported marked subjective visual improvement. Late postoperative capsular bag distension syndrome is an uncommon and often misdiagnosed cause of delayed visual decline after cataract surgery. Recognition of its characteristic slit lamp findings is essential, and Nd:YAG posterior capsulotomy remains a safe and effective treatment, even many years after the initial surgery.

## Introduction

Capsular bag distension syndrome (CBDS), also referred to as capsular block syndrome, is an uncommon complication of cataract surgery with an incidence of less than 1% [1]. It is characterized by the accumulation of fluid within the capsular bag, trapped between the posterior capsule and the intraocular lens (IOL), resulting in capsular bag distension and visual disturbances. Although it may occur intraoperatively or in the early postoperative period, late postoperative CBDS represents a distinct clinical entity that can present several months to years after cataract surgery.

Patients with late postoperative CBDS may experience progressive visual decline due to the accumulation of opaque or turbid fluid behind the IOL. Given the timeline of the presentation of CBDS, it may be misdiagnosed as posterior capsular opacification and thus, awareness of this entity and its characteristic slit lamp findings is essential for timely diagnosis and appropriate management.

## Case presentation

A 74-year-old man presented to the Moran Eye Center with a complaint of progressively worsening hazy vision in his right eye over the past year. His ocular history was notable for cataract extraction with posterior chamber IOL implantation in the right eye approximately 15 years prior. There was no history of ocular trauma or recent intraocular surgery. Prior to his presentation, the patient had been evaluated by an outside ophthalmologist in Alaska, where a neodymium-doped yttrium aluminum garnet (Nd:YAG) posterior capsulotomy had been attempted without success.

On examination at initial presentation, visual acuity was 20/30 in the right eye, correcting to 20/25 with pinhole. Intraocular pressure was within normal range at 16 mm Hg by handheld tonometry, with otherwise normal afferent and efferent examination functions.

**Figure 1:**
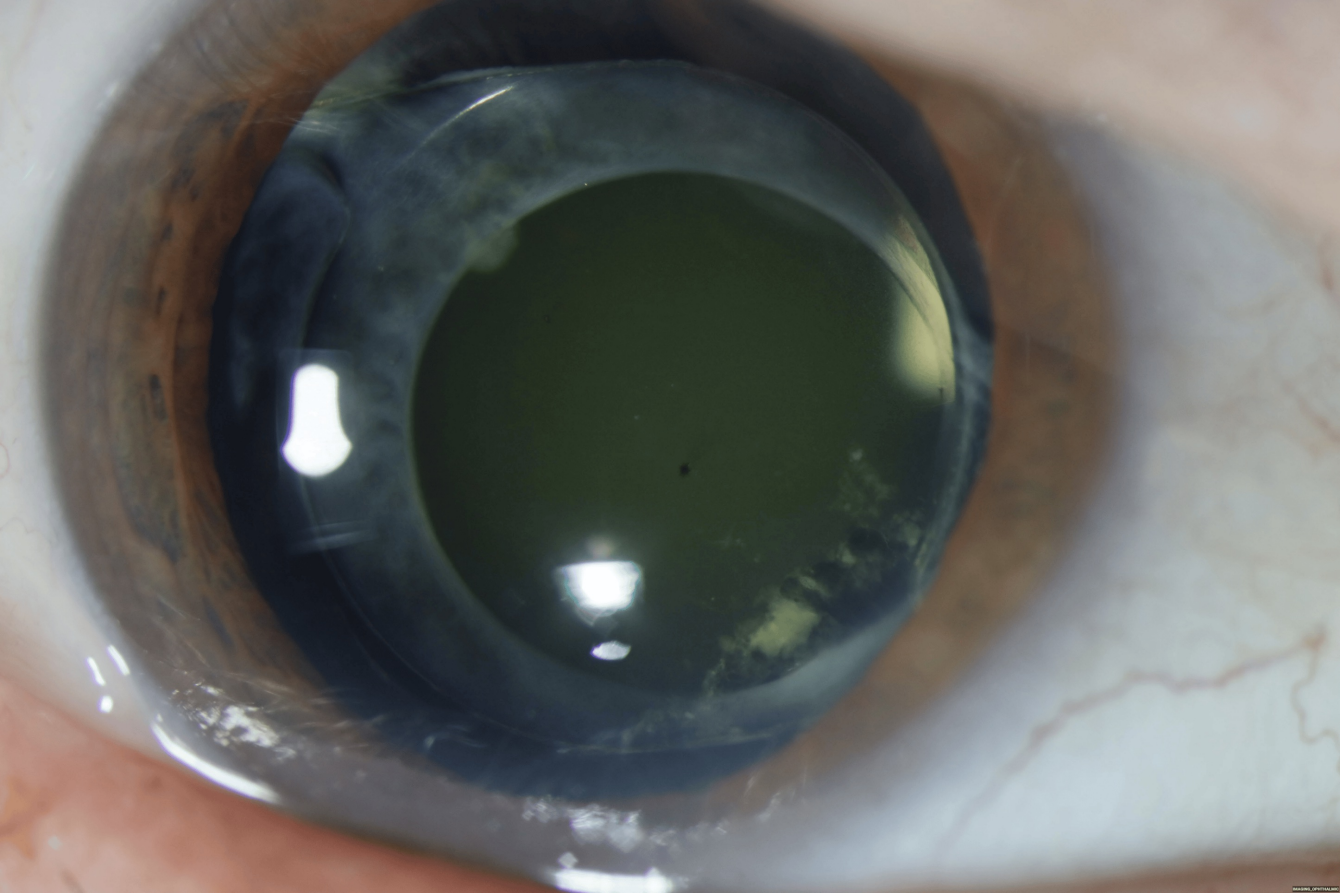
Right eye, diffuse illumination Turbid yellow-white distends the capsular bag behind the intraocular lens.

**Figure 2:**
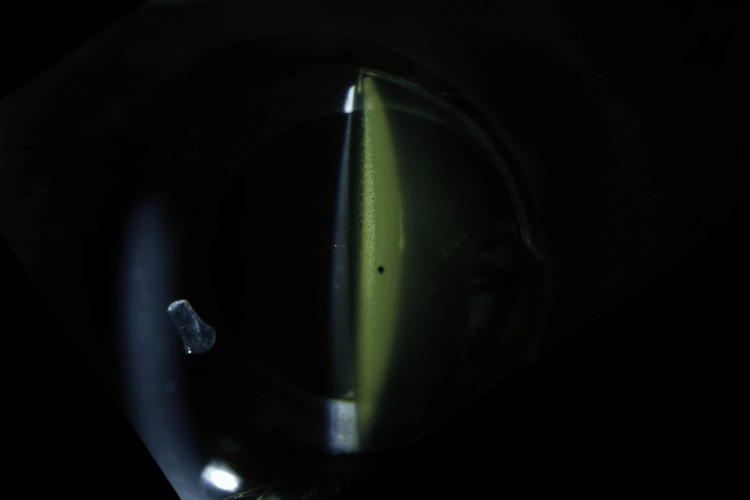
Right eye, narrow beam Slit beam outlines the distended posterior capsule behind the intraocular lens (IOL) optic. Central IOL surface shows a small laser pit consistent with prior neodymium-doped yttrium aluminum garnet (Nd:YAG) posterior capsulotomy attempt.

On slit lamp examination, the right eye demonstrated yellowish turbid fluid between the posterior capsular bag and the IOL optic, with the posterior capsule appearing bowed posteriorly (Figures 1, 2). A small central laser pit was visible on the IOL optic, consistent with the unsuccessful prior Nd:YAG laser attempt. No significant anterior chamber inflammation was noted. Based on the characteristic clinical findings, a diagnosis of late postoperative CBDS was made.

A repeat Nd:YAG posterior capsulotomy was performed at the slit lamp utilizing an Abraham capsulotomy lens to improve the laser’s focus on the posterior capsule. A total of 11 laser shots (20.2 mJ in total) successfully opened the posterior capsule, resulting in immediate decompression of the capsular bag and posterior displacement of the turbid yellow fluid into the vitreous cavity. The IOL remained well-centered with no complications. Prednisolone acetate was prescribed four times daily for one week. The patient's follow-up was planned in three months for repeat slit-lamp photographs and refraction.

The patient was unable to attend in person for his follow-up appointment, and objective postoperative data were therefore unavailable, which is a limitation of this case report. Nevertheless, four months following the procedure, the patient was spoken to over the phone and reported that his vision had drastically improved and was subjectively at the same visual acuity as his left eye.

## Discussion

CBDS refers to a group of related complications after cataract surgery in which fluid becomes trapped within the capsular bag. Late postoperative CBDS may be mistaken for more common causes of late visual decline after cataract surgery, rendering a focused differential diagnosis essential. Posterior capsule opacification is frequently presumed (as in our case initially), but it does not account for a discrete collection of turbid fluid behind the lens or even posterior capsule distension/bowing. Chronic postoperative endophthalmitis (classically associated with Cutibacterium acnes) should be considered, especially when symptoms are accompanied by pain or recurrent intraocular inflammation with an occasional capsular plaque. When turbid intracapsular material is present, this distinction can be diagnostically challenging. However, late CBDS typically occurs in a relatively quiet eye, without anterior chamber inflammation, hypopyon, or pain, despite opaque or milky fluid within the capsular bag. Additional mimickers include IOL optic opacification/glistenings, uveitis-glaucoma-hyphema (UGH) syndrome, retained lens material, and Soemmering ring-related changes.

Miyake and colleagues first proposed a classification system dividing the condition into intraoperative, early postoperative, and late postoperative forms [2]. The late postoperative variant typically presents several years after surgery and is thought to result from proliferation and pseudometaplasia of residual lens epithelial cells, leading to production of proteinaceous or liquefied material within a sealed capsular bag and a functional “closed chamber” [2-4].

Kim and Shin later expanded on this classification, introducing fibrotic, noncellular, and inflammatory subtypes [1]. Late postoperative capsular bag distension syndrome most closely aligns with the fibrotic/non-inflammatory group, which is characterized by opaque or milky fluid accumulation behind the IOL and minimal anterior chamber inflammation, features that help distinguish it from infectious endophthalmitis or uveitis/glaucoma syndromes described previously. Clinically, the diagnosis is often made by slit lamp examination, with characteristic findings including bowing of the posterior capsule and visible turbid or yellow fluid within the capsular bag. Ancillary imaging modalities such as anterior segment optical coherence tomography (OCT) or ultrasound biomicroscopy may aid in diagnosis when visualization is limited.

The first-line approach for late postoperative CBDS is typically Nd:YAG posterior capsulotomy because it opens the posterior capsule and allows the fluid to drain into the vitreous cavity [5]. Multiple reports describe improvement in visual acuity after Nd:YAG posterior capsulotomy in eyes with late postoperative CBDS [6,7]. If visualization of the posterior capsule is limited by dense fluid or poor dilation, a peripheral iridotomy followed by Nd:YAG anterior capsulotomy through the iridotomy to puncture the underlying anterior capsule is an alternative method that has been described in the literature [8]. Femtosecond laser-assisted posterior capsulotomy with intraoperative OCT guidance can be another option when visualization is difficult, which has been reported to safely relieve an opaque late postoperative case [9]. Lastly, pars plana vitrectomy and posterior capsulotomy has also been reported to be an effective option, however, it is much more invasive and carries the risks of intraocular surgery [10].

## Conclusions

In conclusion, this case illustrates a delayed presentation of late postoperative CBDS occurring more than a decade after cataract surgery. Recognition of its distinctive slit lamp findings is critical for accurate diagnosis, particularly in patients presenting with late onset visual decline after cataract surgery. Nd:YAG posterior capsulotomy remains a safe and effective treatment, capable of improving visual acuity with minimal risk when appropriately performed.
